# Genetic evidence for vitamin D and cardiovascular disease: choice of variants is critical

**DOI:** 10.1093/eurheartj/ehab870

**Published:** 2022-05-07

**Authors:** Stephen Burgess, Dipender Gill

**Affiliations:** 1 Medical Research Council Biostatistics Unit, University of Cambridge, Cambridge CB2 0SR, UK; 2 Department of Public Health and Primary Care, University of Cambridge, Cambridge CB1 8RN, UK; 3 Department of Epidemiology and Biostatistics, School of Public Health, Imperial College London, London W2 1PG, UK; 4 Clinical Pharmacology and Therapeutics Section, Institute of Medical and Biomedical Education and Institute for Infection and Immunity, St George’s, University of London, London SW17 0RE, UK; 5 Clinical Pharmacology Group, Pharmacy and Medicines Directorate, St George’s University Hospitals NHS Foundation Trust, London SW17 0QT, UK; 6 Genetics Department, Novo Nordisk Research Centre Oxford, Old Road Campus, Oxford OX3 7FZ, UK

## Abstract

**Graphic abstract:**
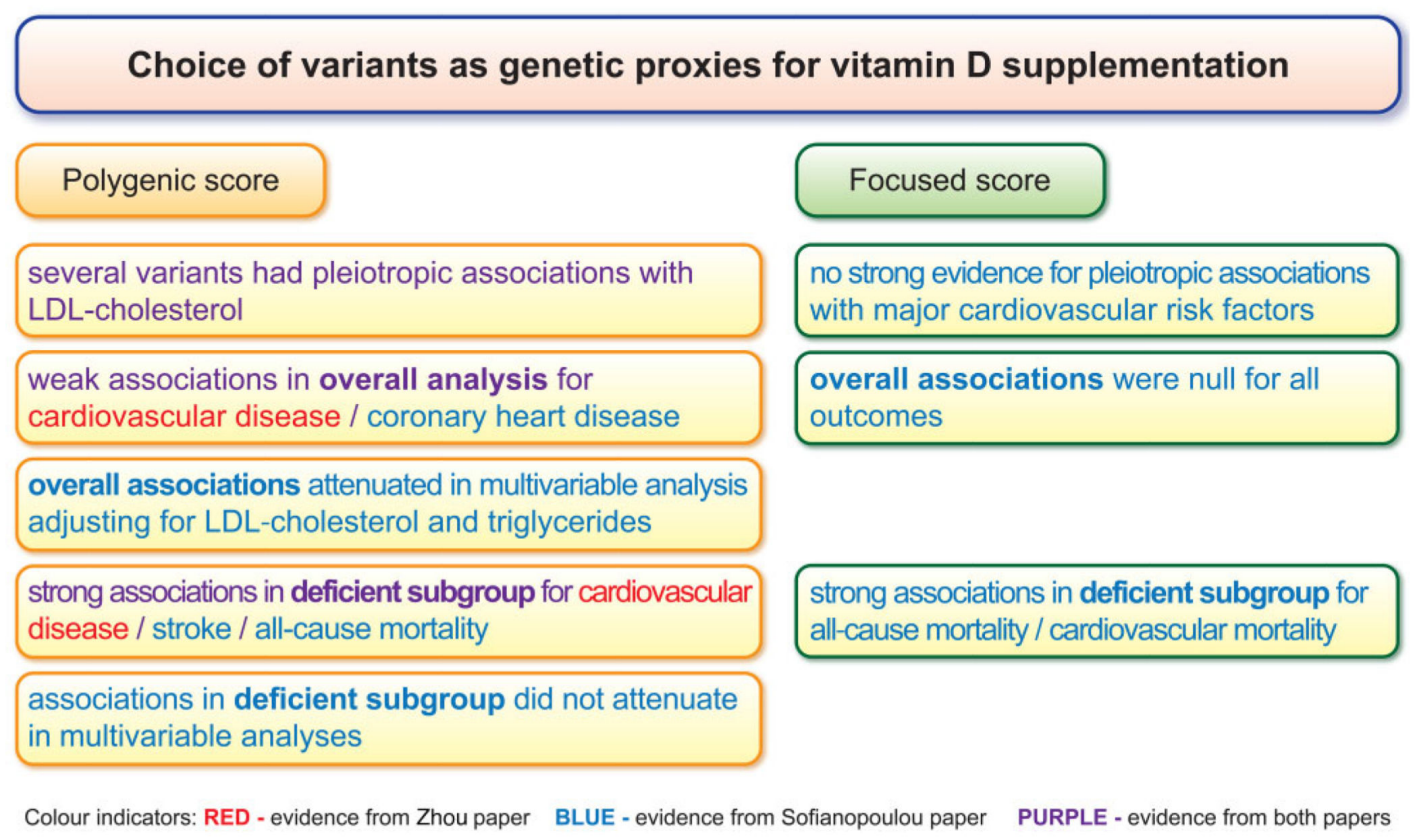
Contrasting findings from Mendelian randomization analyses performed using a polygenic choice of genetic variants (that is, all genome-wide significant predictors of 25-hydroxyvitamin D) to those using a focused choice of genetic variants (that is, variants in four gene regions related to vitamin D synthesis and metabolism).

Contrasting findings from Mendelian randomization analyses performed using a polygenic choice of genetic variants (that is, all genome-wide significant predictors of 25-hydroxyvitamin D) to those using a focused choice of genetic variants (that is, variants in four gene regions related to vitamin D synthesis and metabolism).

Mendelian randomization is an epidemiological technique that compares disease risk in groups of individuals defined based on their genetic variants to make causal inferences.^1^ Rather than comparing individuals with high levels of an exposure vs. those with low levels of an exposure as in a conventional epidemiological analysis, the approach compares those with genetic variants predisposing them to increased vs. decreased levels of the exposure. This is analogous to the analysis of a randomized controlled trial for a treatment that increases levels of the exposure, which does not compare those with high vs. low levels of the exposure after treatment, but rather compares those who were randomly assigned to receive the treatment vs. those who were randomly assigned to the control group. The logic is that randomization should be independent of all competing risk factors, meaning that the randomly assigned groups only differ systematically with respect to their average levels of the treatment and any downstream consequences of the treatment. Hence, an association between randomization and the trial outcome is indicative of a causal effect of the treatment. In the same way, if genetic variants act analogously to randomization by dividing the population into groups that differ systematically only with respect to the exposure and its consequences, then an association between the genetic variants and the outcome is indicative of a causal effect of the exposure.^2^

Non-linear Mendelian randomization is an extension to standard Mendelian randomization that first stratifies the population based on levels of the exposure, and then conducts separate Mendelian randomization analyses within each stratum.^3,4^ One important methodological point is that stratifying on the exposure directly would break randomization and lead to biased estimates in the strata.^5^ This is because the distribution of the genetic variants would no longer be the same within each stratum, as genetic variants predisposing individuals to higher levels of the exposure would be more common in strata with high levels of the exposure and less common in strata with low levels of the exposure. For this reason, it is recommended to first regress the exposure on the genetic variants, and stratify on residual values of the exposure, as this ‘residual exposure’ is independent of the genetic variants, and hence randomization still holds within strata of the residual exposure.^3,4^

In their study published in this issue of the *European Heart Journal*, Zhou *et al*. considered genetic predictors of 25-hydroxyvitamin D [25(OH)D], a clinical biomarker used to measure vitamin D status, and assessed whether these variants were associated with cardiovascular disease risk in individuals of European genetic ancestry from the UK Biobank dataset.^6^ They observed inverse associations in overall analyses, but particularly strong inverse associations in non-linear analyses for strata of the population with low levels of 25(OH)D. The non-linear analyses indicated a threshold-like relationship, suggesting greater benefit of vitamin D supplementation for those who are vitamin D deficient, and less benefit for those with adequate vitamin D levels.

The results are similar to those recently published by Sofianopoulou *et al*., which showed threshold relationships for allcause and cardiovascular mortality in individuals of European genetic ancestry from the UK Biobank dataset (as well as three smaller datasets), with evidence supporting a beneficial effect of higher 25(OH)D in individuals that are vitamin D deficient up to a threshold of ~40 nmol/L, but with no evidence of benefit when considering the population as a whole.^7^ Although they used broadly the same data, there are two major methodological differences between these studies. First, Zhou *et al*. performed a polygenic analysis considering 35 genetic variants from multiple gene regions (referred to as a ‘polygenic score’), whereas Sofianopoulou *et al*. considered variants from four gene regions that have biological links to vitamin D synthesis and metabolism (referred to as a ‘focused score’). Secondly, Zhou *et al*. considered a wider outcome definition, incorporating coronary artery disease, stroke, and peripheral vascular disease, whereas Sofianopoulou *et al*. considered coronary heart disease and stroke as separate outcomes, as well as cardiovascular mortality. Analyses in the study of Sofianopoulou *et al*. for coronary heart disease and stroke provided null associations overall, and inverse associations in the deficient stratum, but with less statistical precision arising from the lower number of events due to considering the outcomes separately. Table 1 summarizes differences in the methodology and results between the studies.

One concern in the study of Zhou *et al*. is potential pleiotropy via LDL-cholesterol, which is implicated in cardiovascular disease pathophysiology. While the authors conducted analyses to assess pleiotropic associations with several covariates, they did not specifically consider LDL-cholesterol. Taking genetic associations in White British participants from the UK Biobank reported by Neale *et al*.,^8^ three of the 35 variants in the study of Zhou *et al*. were associated with LDL-cholesterol at *P* < 5 x 10^−8^ (rs7528419, rs78151190, and rs261291), and a further four variants were associated at *P* < 0.005 (rs6671730, rs6123359, rs17216707, and rs2585442). A polygenic score was also considered in the study of Sofianopoulou *et al*. as a secondary analysis; however, this analysis was also affected by pleiotropy via LDL-cholesterol. The polygenic analysis of Sofianopoulou *et al*. found an overall association with coronary artery disease that attenuated in a multivariable Mendelian randomization model adjusting for genetically predicted LDL-cholesterol and triglyceride levels. However, evidence for an inverse association with stroke in vitamin D-deficient individuals in the polygenic analysis persisted despite the same adjustment; while the estimate in the deficient stratum attenuated on adjustment, evidence for an inverse association remained. This suggests that associations indicated by polygenic analyses in the deficient stratum may be robust to pleiotropy, but associations in the overall population may not be robust to pleiotropy via LDL-cholesterol.

A further potential concern with the study of Zhou *et al*. is the presentation of population impact factors. Genetic estimates in Mendelian randomization represent differences in disease risk between groups having lifelong differences in the distribution of the exposure.^9^ Replicating such estimates in practice would require lifelong interventions to increase 25(OH)D levels. Hence the achievable population benefit for cardiovascular risk reduction through vitamin D supplementation is likely to be overestimated.^10^ There are also potential harms of high levels of vitamin D supplementation for those with already adequate levels of 25(OH)D.11 These factors suggest that any clinical trial to demonstrate benefits of vitamin D supplementation should be large in size, focused on a vitamin D-deficient population, and have a long follow-up period. Contamination by vitamin D supplementation in the control group would further potentially limit the effectiveness of the trial. Such a trial may therefore be difficult to implement in practice.

Overall, the finding of the study of Zhou *et al*. supporting that vitamin D supplementation reduces risk of cardiovascular disease for vitamin D-deficient individuals is plausible, and is supported by the existing literature.^7^ The association of genetically predicted levels of 25(OH)D with cardiovascular disease risk estimated by Zhou *et al*. in deficient individuals is so large in magnitude that it is unlikely to be wholly attributable to pleiotropic associations of the genetic variants in the analysis. In contrast, the notion that vitamin D reduces risk of cardiovascular disease in individuals with adequate levels of 25(OH)D is more questionable, as findings in the genetic analyses could be fully explained by pleiotropic associations of variants with LDL-cholesterol. Generally speaking, it is recommended that Mendelian randomization studies consider a biologically informed choice of genetic variants where possible, and, if not possible, consider pleiotropic associations with key determinants of disease risk.^12^ For cardiovascular disease, this should typically include LDL-cholesterol. For this reason, we would favour Mendelian randomization analyses with vitamin D as an exposure that use a more focused set of genetic variants, rather than analyses that include all genome-wide significant predictors of 25(OH)D.

In conclusion, this investigation strengthens the evidence base for vitamin D supplementation as a beneficial intervention to reduce cardiovascular disease risk in individuals with low vitamin D levels. Future research should aim to consider other disease outcomes and population groups, particularly those with low average levels of vitamin D.

## Figures and Tables

**Table 1:** Differences between the studies of Zhou and Sofianopoulou in methodology and results

Factor	Zhou et *al*.^6^	Sofianopoulou et *al*.^7^
Choice of variants	35 variants associated with 25(OH)D levels at genome-wide significant level	Variants in 4 gene regions chosen due to biological link with vitamin D synthesis or metabolism (‘focused score’); a secondary analysis using 71 genome-wide significant variants was also performed
Pleiotropic associations	Several genetic variants had pleiotropic associations with LDL-cholesterol	Minimal evidence of pleiotropy for primary analysis; substantial pleiotropy via LDL-cholesterol and triglycerides for secondary analysis using genome-wide variants
Cardiovascular outcomes	Cardiovascular disease (combined outcome of coronary artery disease, stroke, and peripheral vascular disease)	Separate analyses for coronary heart disease, stroke, and cardiovascular mortality
Results	Strong associations of genetically predicted 25(OH)D with outcomes in vitamin D-deficient strata; weak (but non-null) associations overall	**Primary analysis:** associations of genetically predicted 25(OH)D with outcomes in vitamin D-deficient strata (particularly for cardiovascular mortality); null associations overall **Secondary analysis:** weak association with coronary heart disease in overall analysis that attenuated toward null on adjustment for LDL-cholesterol and triglycerides; stronger association with stroke in deficient stratum that persisted despite adjustment for LDL-cholesterol and triglycerides
